# Electroacupuncture alleviates neuromuscular dysfunction in an experimental rat model of immobilization

**DOI:** 10.18632/oncotarget.20246

**Published:** 2017-08-14

**Authors:** Jun Yang, Su Min, Fei Xie, Jingyuan Chen, Xuechao Hao, Li Ren

**Affiliations:** ^1^ Department of Anesthesiology, The First Affiliated Hospital of Chongqing Medical University, Chongqing 400016, China

**Keywords:** electroacupuncture, nicotinic acetylcholine receptors, immobilization, skeletal muscle atrophy, neuromuscular function

## Abstract

Immobilization-related skeletal muscle atrophy is a major concern to patients in Intensive Care Units and it has a profound effect on the quality of life. However, the underlying molecular events for the therapeutic effect of electroacupuncture to treat muscle atrophy have not been fully elucidated. Here we developed an immobilization mouse model and tested the hypothesis that skeletal muscle weakness may be caused by the increased expression of γ and α7 nicotinic acetylcholine receptors (nAChRs) on muscle cell membranes, while electroacupuncture could decrease the expression of γ and α7 nicotinic acetylcholine receptors. Compared with the rats in control, those treated with immobilization for 14 days showed a significant reduction of tibialis anterior muscle weight, muscle atrophy and dysfunction, which was associated with a significant decrease expression of neuregulin-1 and increased expression of γ- and α7-nAChR in tibialis anterior muscle. Electroacupuncture significantly enhanced the expression of neuregulin-1 and alleviated the muscle loss, while diminished the expression of γ- and α7-nAChR. Taken together, the beneficial effect of electroacupuncture may be attributed to suppressing γ- and α7-nAChR production, enhancing neuromuscular function and neuregulin-1 protein synthesis. These results suggest that electroacupuncture is a potential therapy for preventing muscle atrophy during immobilization.

## INTRODUCTION

Muscle atrophy is one of the most important and frequent problems observed in patients in Intensive Care Units [1]. The immobilization therefore is a factor that helps the development of muscular atrophy [2]. In immobilization-related disorders or pathological states, there is loss of muscle mass and decreased muscle strength [3, 4]. Immobilization changes the function as well as structure of the peripheral neuromuscular system, and alters both protein degradation and synthesis [5]. These have significant clinical consequences, up to and including excess morbidity and mortality. Therefore, effective therapeutic strategies to treat muscle wasting induced by immobilization are urgently required.

Acupuncture is a branch of traditional Chinese medicine that is widely applied to treat various diseases around the world [6, 7]. Electroacupuncture (EA) is an acupuncture technique that replicates the benefits of exercise through stimulation of muscle contraction. EA has been shown to decrease skeletal muscle atrophy induced by hind-limb suspension in mice [8]. Previous studies have found that EA is a non-pharmacologic approach that can prevent muscle loss induced by chronic kidney disease [9]. The mechanism of EA has been extensively investigated, including increases oxygenation of skeletal muscles and down-regulates the expression of ubiquitinated proteins [10–12]. The mechanism of EA on muscle atrophy, however, has not yet been sufficiently elucidated.

The neuronal α7-type nicotinic acetylcholine receptor (α7-nAChR) and the fetal-type (γ-nAChR) mRNA encoding could be increased after immobilization has been confirmed [13–15]. In the early fetal stage, the α7-nAChR and γ-nAChR, were scattered throughout the muscle membrane before innervation, while only ε-nAChR was synthesized in muscle cells and was anchored to the end-plate in adults [16]. Our previous study has been shown that the re-expression of γ- and α7-nAChR in mature neuromuscular junction (NMJ) leads to dysfunction and pharmacodynamics changes in non-depolarizing muscle relaxant(s) (NDMRs) in the septic state [17]. However, it remains unknown whether the neuromuscular dysfunction and muscle atrophy induced by immobilization is associated with the re-expression of the γ- and α7-nAChR in skeletal muscle.

Neuregulin-1 (NRG-1) is a multifunctional protein, it played a significant role in peripheral nerve regeneration and remyelination [18]. Recent studies have established that neuromuscular function was associated with NRG-1 in the sepsis model [19]. Following nerve injury, Schwann cells responds to axonal damage up-regulating NRG-1 and improves remyelination. The NRG-1 plays an important role in this process, thanks to their ability to stimulate proliferation and differentiation of Schwann cells *in vitro* [20, 21].

In this study, we aimed to test the hypothesis that immobilization could decrease the neuromuscular function by increasing γ- and α7-nAChR expression in the skeletal muscle. We used a typical disuse model that was induced by pining-immobilization to examine the effect of loss of skeletal muscle mass and the changes in the composition of the nAChRs. We also assessed neuromuscular function with hierarchical cluster analysis, principal component analysis and in relation to the expression of γ- and α7 subunits of acetylcholine receptors. EA would enhance the NRG-1 signaling pathway, and decrease γ- and α7-nAChR expression, resulting in suppressed immobilization- induced muscle loss and alleviated neuromuscular dysfunction. The positive effects of EA could provide an additional therapeutic option for treatment of muscle atrophy.

## RESULTS

### EA prevents immobilization-induced muscle fiber cross-sectional area and muscle fiber size decrease

**Figure 1:**
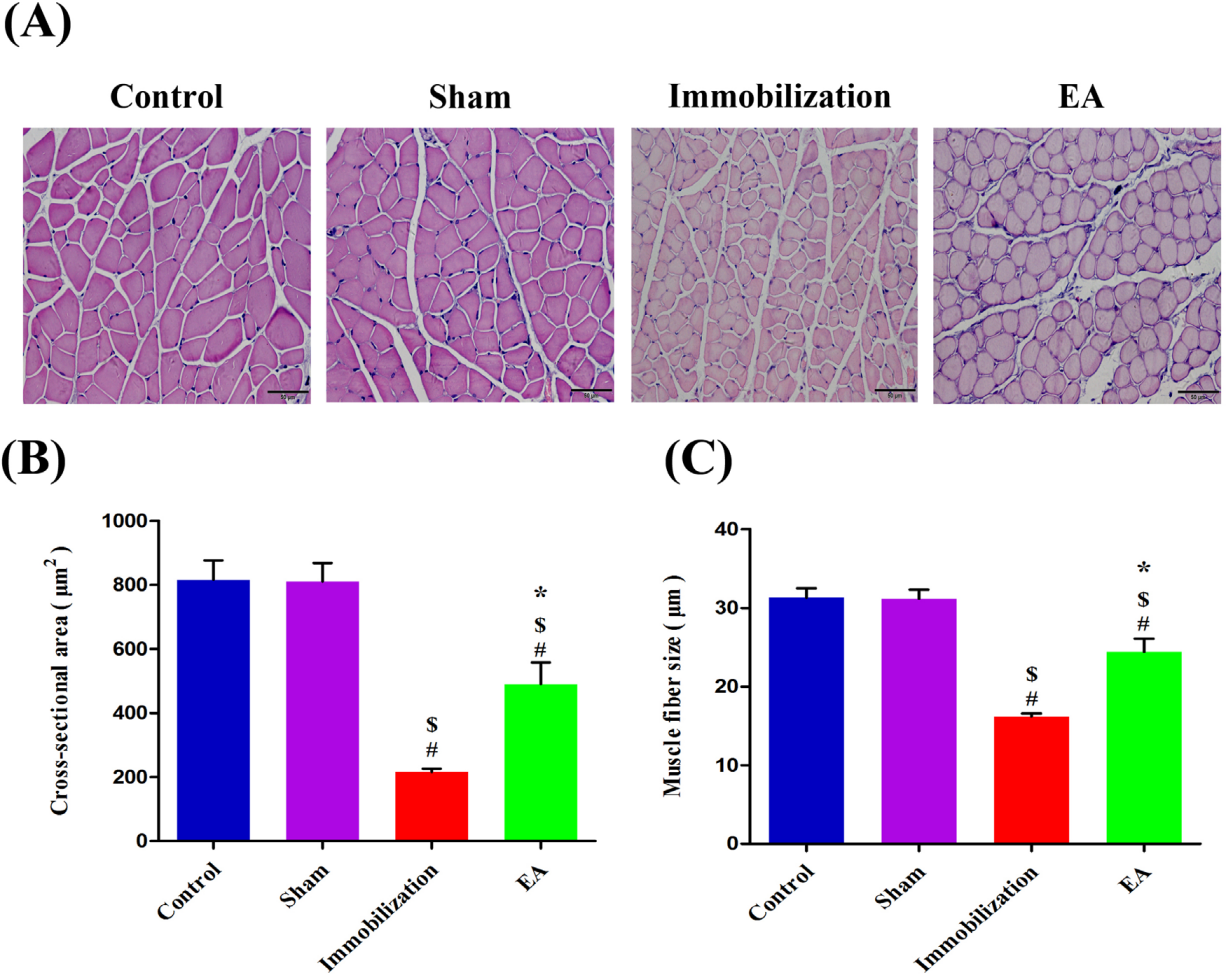
EA prevents immobilization-induced muscle fiber cross-sectional area and muscle fiber size decrease **(A)** The morphological changes of tibialis anterior muscle stained with H&E (magnification, ×200, scale bar = 50μm) in four groups. **(B**, **C)** After 14 days immobilization, muscle fiber Cross-sectional area andmuscle fiber size were significantly decreased in the Immobilization group than in the Control group (*P* < 0.05). With EA treatment, Cross-sectional area andmuscle fiber size were significantly greater in the EA group than in the Immobilization group (*P* < 0.05). The average size of myofibers determined from six mice x 10 sections/mouse/group (Bars: means ± SD; n = 6/group). *P* < 0.05 is significant (# vs. Control, $ vs. Sham, *vs. Immobilization).

Muscle fiber cross-sectional area was determined in paraffin sections of tibialis anterior muscles using an H&E staining (Figure 1A). After 14 days immobilization, muscle fibercross-sectional areaand muscle fiber size were significantly reduced in Immobilization group compared with Control group (73% and 48% reductions, respectively) (Figure 1B and 1C). Furthermore, immobilization-induced reduction in the cross-sectional area and muscle fiber size was significantly improved in the EA group (127% and 51% increase, respectively) (Figure 1B and 1C). EA suppressed the decrease in the muscle fibercross-sectional area and muscle fiber size.

### EA prevents immobilization-induced muscle atrophy

**Figure 2:**
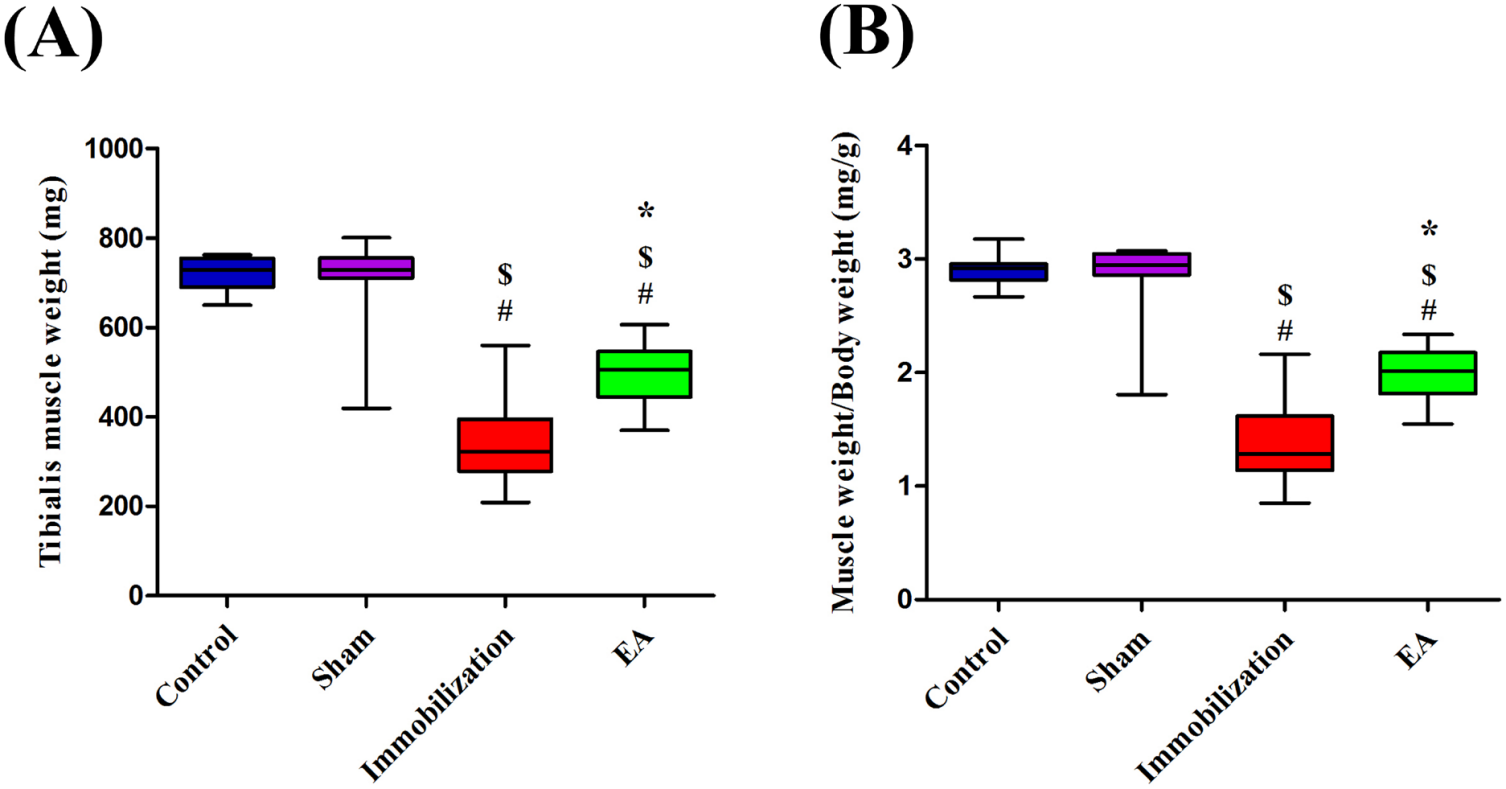
EA prevents immobilization-induced muscle atrophy **(A)** Box plot shows Tibialis anterior muscle weight. **(B)** Box plot shows muscle wet weight/body weight ratio at different groups. (n=8/group). ^#^ *P* < 0.05 vs. Control group; ^$^ *P* < 0.05 vs. Sham group; ^*^ *P* < 0.05 vs. Immobilization group.

The weights of tibialis anterior muscles in rats with immobilization were significantly less than those of control rats (Immobilization: 322.6 [278.5, 395.3] mg; Control: 729.3 [690.2, 754.3] mg). However, the tibialis anterior muscle weights were significantly higher in rats with EA treatment than in rats with immobilization (EA: 505.9 [444.8, 546.2] mg; Immobilization: 322.6 [278.5, 395.3] mg) (Figure 2A). Moreover, the values of muscle wet weight/body weight ratio in the Control and Immobilization group showed the similar trend as the muscle weight. Compared with Immobilization group, EA group have a higher muscle wet weight/body weight ratio (EA: 2.0 [1.8, 2.2], Immobilization: 1.3 [1.1, 1.6]) (Figure 2B). All of this indicated that immobilization induced muscle atrophy and EA treatment prevented immobilization-induced muscle wasting.

### EA improves neuromuscular function

**Figure 3:**
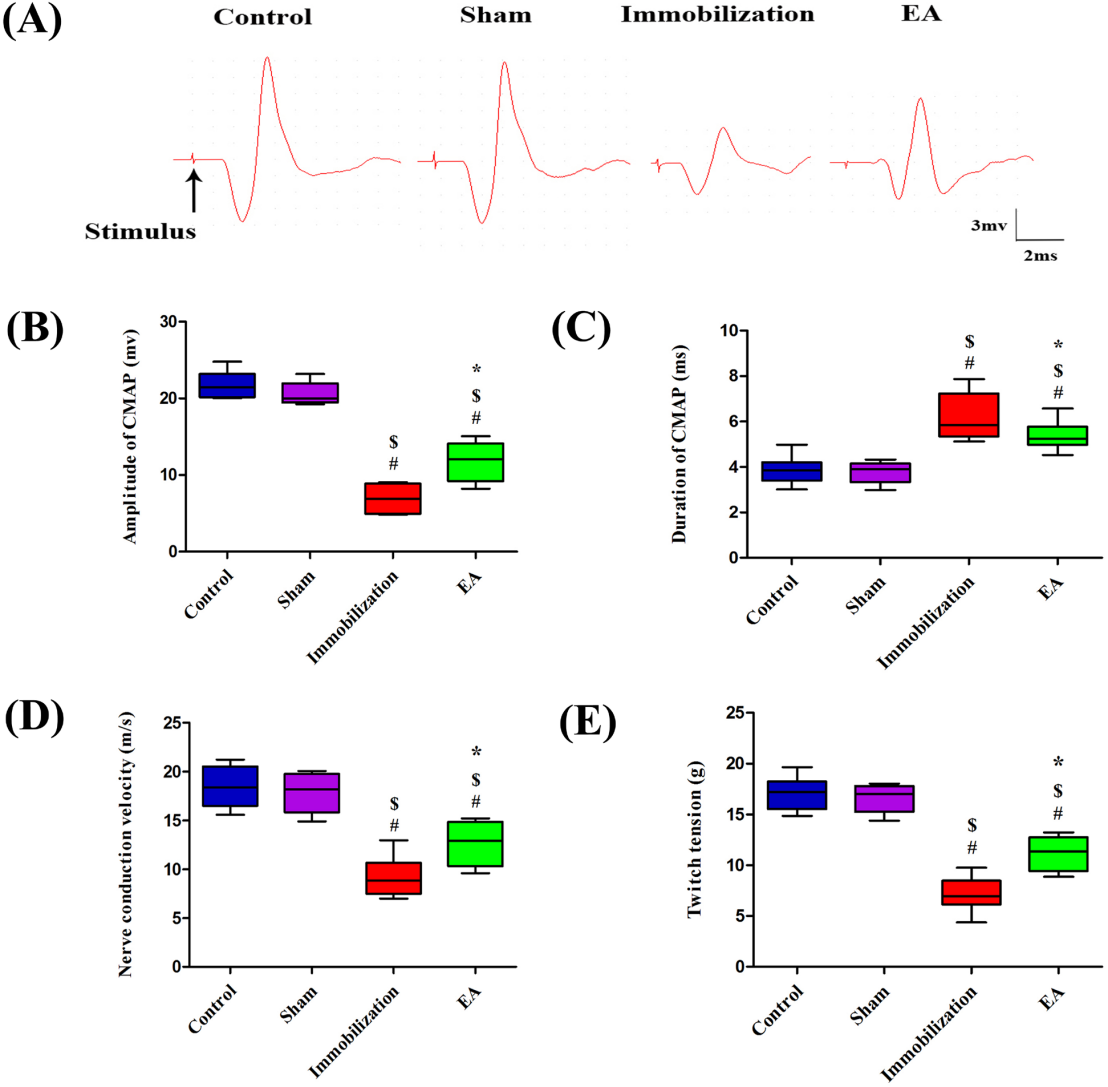
EA improves neuromuscular function CMAP recorded in the tibialis anterior muscle by needle electrodes after supramaximal needle electrode stimulation of the sciatic nerve. **(A)** Samples of CMAP recorded in each group, **(B)** amplitudes, **(C)** durations, **(D)** NCV, and **(E)** single-twitch tension elicited by continuous indirect stimulation. (n=8/group). ^#^ *P* < 0.05 vs. Control group; ^$^ *P* < 0.05 vs. Sham group; ^*^ *P* < 0.05 vs. Immobilization group.

The compound muscle action potential (CMAP) recording of each group was shown in Figure 3A. Amplitude, duration, NCV and twitch tension of CMAP in Control group exhibited no significant difference compared with Sham group. After 14 days of immobilization, amplitude, NCV and twitch tension of CMAP decreased significantly compared with control group (Control: 21.5 [20.2, 23.2] mv, 18.4 [16.5, 20.5] m/s, 17.2 [15.5, 18.2] g, Immobilization: 6.9 [4.9, 8.8] mv, 8.8 [7.5, 10.6] m/s, 6.9 [6.1, 8.5] g). However, with EA administration, immobilization-induced reduction in amplitude, NCV and twitch tension were significantly improved in the EA group (Immobilization: 6.9 [4.9, 8.8] mv, 8.8 [7.5, 10.6] m/s, 6.9 [6.1, 8.5] g, EA: 12 [9.2, 14.1] mv, 12.9 [10.3, 14.8] m/s, 11.4 [9.4, 12.7] g) (Figure 3B, 3D and 3E). Furthermore, compared with control group, duration of CMAP in Immobilization group prolonged significantly (Control: 3.8 [3.4, 4.2] ms, Immobilization: 5.8 [5.3, 7.2] ms). But EA decreased the duration of CMAP compared with Immobilization group (Immobilization: 5.8 [5.3, 7.2] ms, EA: 5.2 [4.9, 5.7] ms) (Figure 3C). All of this results suggested EA significantly improves neuromuscular function.

### Hierarchical cluster analysis (HCA)

**Figure 4:**
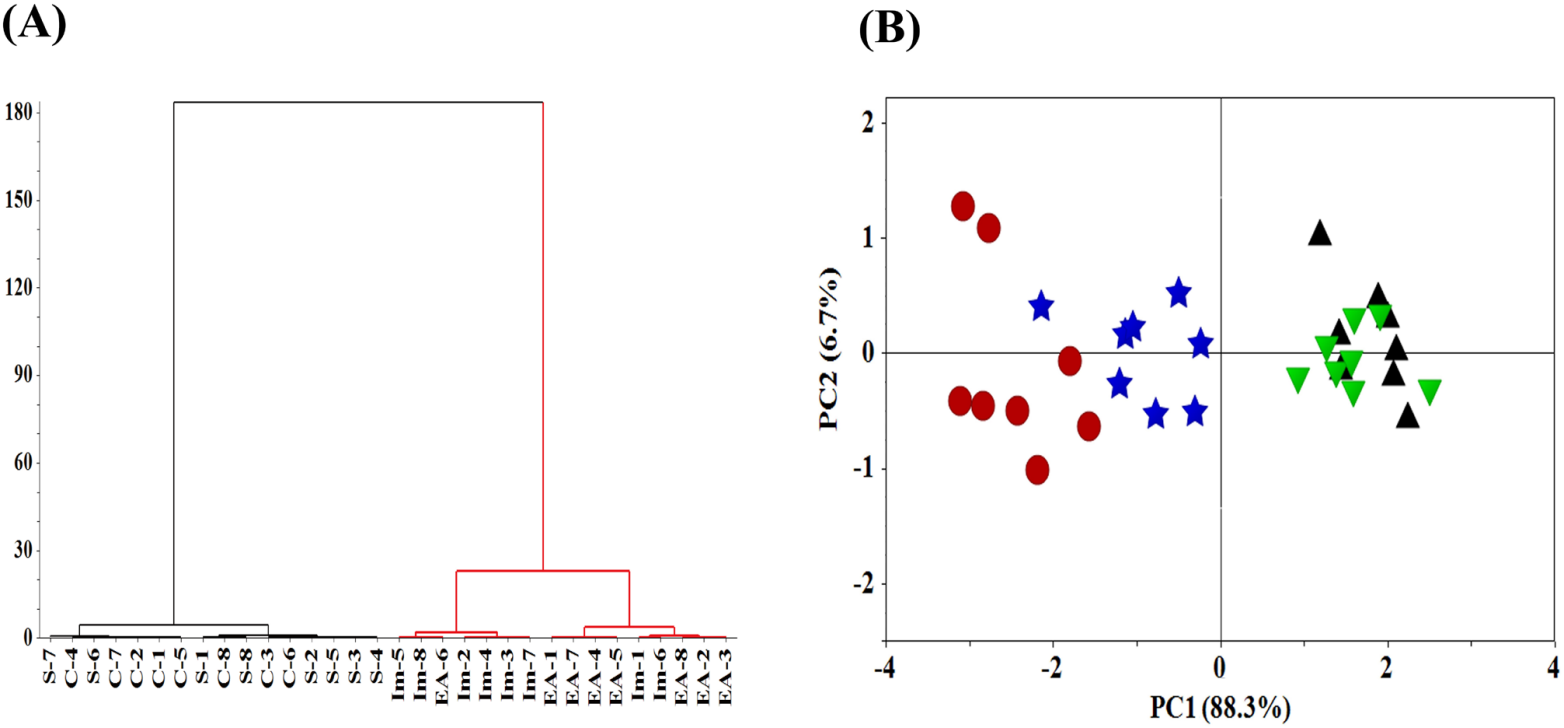
HCA and PCA analysis of neuromuscular function **(A)** HCA, Hierarchical cluster analysis of the neuromuscular function of rats. The neuromuscular function of rats in the Control, Sham, Immobilization and the EA group was labeled as C1∼C8, S1∼S8, Im1∼Im8 and EA1∼EA8. **(B)** PCA scores of neuromuscular function of rats. (▲, Control group; ▼, Sham group; ●, Immobilization group; ★, EA group; n=8/group).

The result of the HCA is shown in Figure 4A. This dendrogram suggests four main clusters (Control-C, Sham-S, Immobilization-Im, EA-EA) with the linkage distance of 0.350, 1.618, 0.925 and 13.05, respectively. The cluster of the Control group and Sham group is very similar with the distance of 3.050, and the next is the Immobilization group and EA group with the distance of 13.05, respectively. With increasing distance between units, successive branch points represent clusters of increasing size and dissimilarity. The distance between the (Control + Sham) group and the (Immobilization + EA) group is 116.8 showing a stronger separation tendency of these groups. Therefore, the normal and pathological conditions of neuromuscular function in rats of muscle atrophy could be separated by CMAP data.

### Principal component analysis (PCA)

The neuromuscular function in rats contains four-dimensional parameters of amplitude, duration, NCV and twitch tension, therefore the PCA model is applied to reduce dimension and obtain an overview of the data. Figure 4B shows the PCA scores of neuromuscular function of rats. The PC1 and PC2 accounts for 88.3% and 6.7% of the total variances, respectively. PC1=0.506×amplitude-0.480×duration+0.508×NCV+0.510×twitch tension. PC2=0.416×amplitude+0.797×duration-0.090×NCV+0.429×twitch tension. The PCA model shows a clear separation tendency of neuromuscular function of rats in the Immobilization group and the Control group. Meanwhile, neuromuscular function of rats in EA group was distinguished from Immobilization group with the EA treatment indicating EA alleviated immobilization-induced neuromuscular dysfunction. The obtained results, therefore, support the fact that CMAP data can be used to differentiate normal and pathological conditions of neuromuscular function of rats in muscle atrophy.

### EA suppresses the expression of α7-nAChR and γ-nAChR in skeletal muscle of immobilization rats

**Figure 5:**
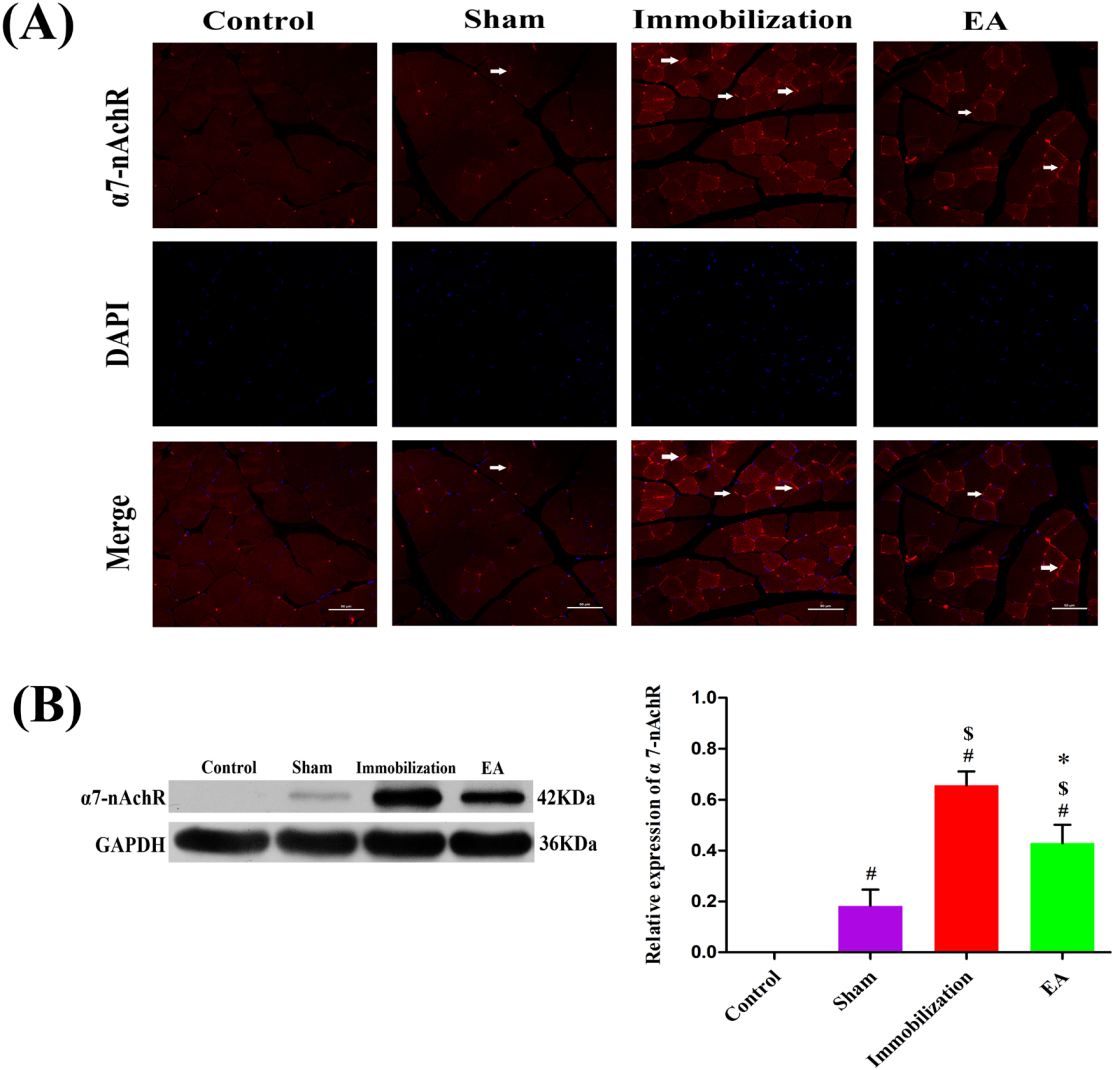
Effects of EA on the expression of α7-nAChR **(A)** Immunofluorescence analysis was performed to determine α7-nAChR expression on Tiabilis anterior muscle 14 days after the procedure. The samples were immunostained with an anti-α7-nAChR-antibody (red, → marks a positive expression). The result shows that α7-nAChR expression are sharply increased and clustered on the muscle membrane of rats in the immobilization group. Representative results from three independent experiments are shown here (scale bar = 50 μm). **(B)** The Western blot analysis of the α7-nAChR proteins are shown for each groups. Relative intensity of α7-nAChR to GAPDH is shown in the graphs. α7-nAChR significantly increased after the immobilization for 14 days. Electroacupuncture suppressed the expression of α7-nAChR in EA group. All values are expressed as means ± SD (n=6/group).^#^ *P* < 0.05 vs. Control group; ^$^ *P* < 0.05 vs. Sham group; ^*^ *P* < 0.05 vs. Immobilization group.

**Figure 6:**
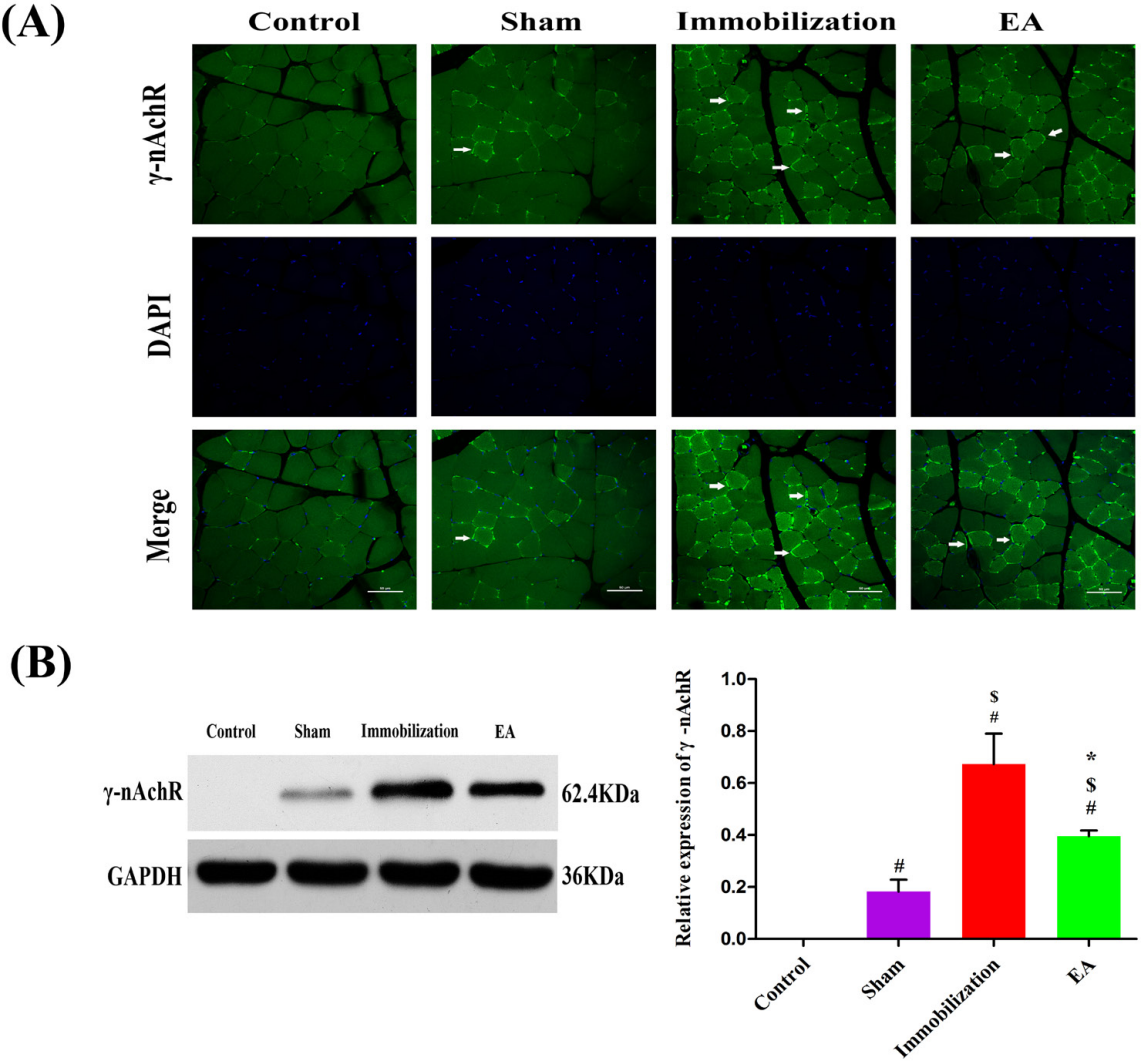
Effects of EA on the expression of γ-nAChR (**A)** Immunofluorescence analysis was performed to determine γ-nAChR expression on Tiabilis anterior muscle 14 days after the procedure. The samples were immunostained with an anti-γ-nAChR-antibody (green, → marks a positive expression). The result shows that γ-nAChR expression are sharply increased and clustered on the muscle membrane of rats in the immobilization group. Representative results from three independent experiments are shown here (scale bar = 50μm). **(B)** The Western blot analysis of the γ-nAChR proteins are shown for each groups. Relative intensity of γ-nAChR to GAPDH is shown in the graphs.γ-nAChR significantly increased after the immobilization for 14 days. Electroacupuncture inhibited the expression of γ-nAChR in EA group. All values are expressed as means ± SD (n=6/group).^#^ *P* < 0.05 vs. Control group; ^$^ *P* < 0.05 vs. Sham group; ^*^ *P* < 0.05 vs. Immobilization group.

The immunofluorescence staining results are shown in Figure 5A and Figure 6A. In control muscle specimens, α7-nAChR and γ-nAChR staining were not observed in the skeletal muscle membrane. In the sham group, the skeletal muscle membrane showed up-regulation of α7-nAChR and γ-nAChR. A great quantity of α7-nAChR and γ-nAChR accumulated on the entire tibialis anterior muscle membrane after immobilization for 14 days. With EA administration, the expression levels of α7-nAChR and γ-nAChR decreased in the skeletal muscle membrane.

The Western blot analysis results are summarized in Figure 5B and Figure 6B. The protein levels of α7-nAChR and γ-nAChR relative to GAPDH significantly increased on immobilization group compared with those in the control and sham group (*P* < 0.05). However, the expression of α7-nAChR and γ-nAChR in the tibialis anterior muscle was significantly decreased in the EA group compared with immobilization group (*P* < 0.05), although these levels of improvement were insufficient when compared with the control group.

### EA increases the expression of NRG-1 in skeletal muscle of immobilization rats

**Figure 7:**
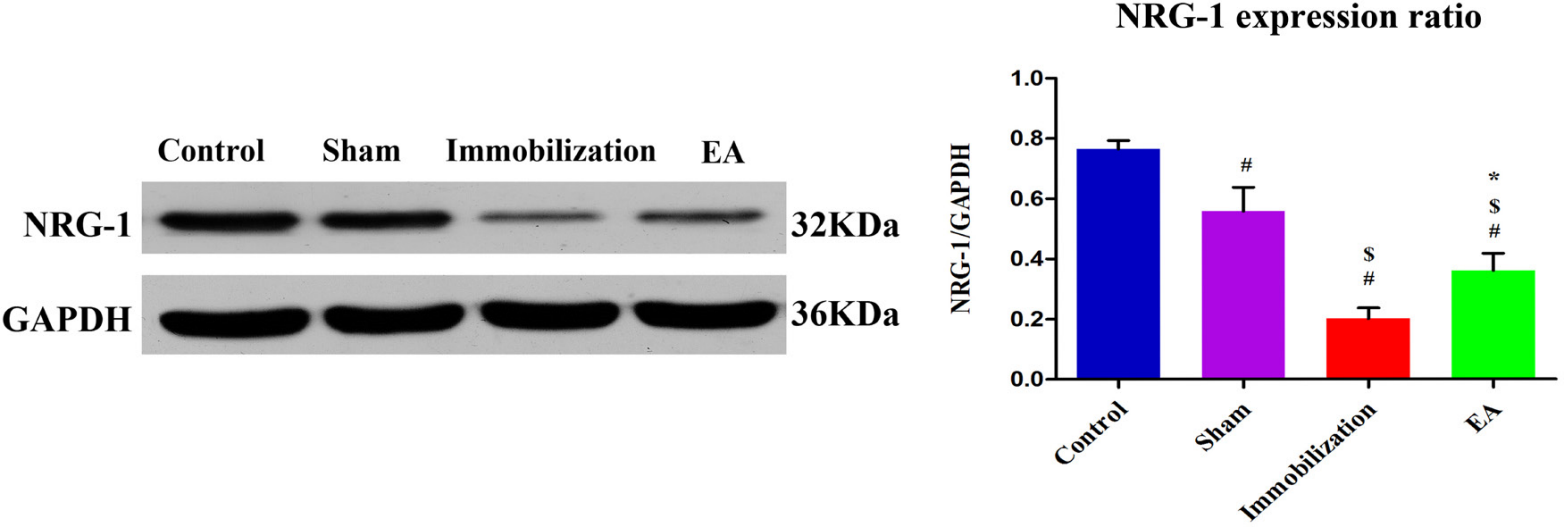
EA activates NRG-1 synthesis in the tiabilis anterior muscle of immobilization rats Western blot analysis indicated that NRG-1 protein levels in the disuse muscles significantly decreased after 14 days of immobilization compared with the control group (*P* < 0.05). Densitometric analysis showed a significant increase in NRG-1 in the tiabilis anterior muscle after electroacupuncture therapy compared with the immobilization group (*P* < 0.05). The integrated optical density of NRG-1 normalized to integrated optical density of GAPDH. The data are shown as means ± SD (n=6/group).^#^ *P* < 0.05 vs. Control group; ^$^ *P* < 0.05 vs. Sham group; ^*^ *P* < 0.05 vs. Immobilization group.

Turning to the expression of protein NRG-1 in tibialis anterior muscle, the expression level of NRG-1 was significantly decreased in the immobilization group compared with the control group and sham group (*P* < 0.05), However, NRG-1 was expressed at higher level in EA group compared with the immobilization group according to Western blot analysis (*P* < 0.05). The results are shown in Figure 7.

## DISCUSSION

In the current study, we showed that the immobilization induces functional and biochemical changes in skeletal muscle of rats. Immobilization would induce muscle atrophy, and the muscle weakness is associated with increased expression of γ- and α7-nAChR. These changes and characteristics are similar to those in the septic model, including re-expression of immature nAChR, reduction of NRG-1, and decrease of CMAP in tibialis anterior muscles [11, 13]. The mechanism of the preventive effect of the EA on muscle atrophy may be attributed to suppressing γ- and α7-nAChR production, enhancing neuromuscular function and NRG-1 protein synthesis.

The rodent model of immobilization has been described previously in rats [22–24], and most recently in mice [25]. Previous methods used to study muscle atrophy have been useful [26, 27]. However, it would be emphasized that only the immobilization model produced by fixation of joints closely replicates the restricted mobility of critical illness people, reduced neuromuscular activity and function changed. Our study has documented the progressive loss of muscle mass with time and the decrease of twitch tension.

The electromyographic examination is a technique for evaluating and recording the electrical activity produced by the skeletal muscle. It is widely used to detect pathophysiology associated with skeletal muscle disorders. Previous studies have shown that disuse weakens aggregate electromyographic activity [28, 29]. In the present study, reduced NCV and prolonged duration time of CMAP were observed 14 days after experimental immobilization. The decline of twitch tension magnitudes elicited by continuous stimulation is concordant with changes of CMAP parameters. Additionally, the HCA and PCA models also demonstrated the normal and pathological conditions of neuromuscular function in rats of muscle atrophy could be separated by CMAP data.

In previous studies, both denervation and immobilization led to the loss of muscle mass and increase in total AChRs on the muscle membrane [30, 31]. As early as the late 1990s, Ibebunjo, C and Nosek, M. T found that immobilization changes the subunits of nAChR, with new nAChR (γ-nAChR) appearing on the muscle membrane [15]. Recently, Khan, M. A and Sahani, N have confirmed that the α7-nAChRs were up-regulated 3-fold along with α1AChRs on the immobilized side as compared with the contralateral side after 21 days of immobilization [14], but they failed to identify a link to the altered nAChRs expression. Interestingly, our findings reveal that the γ- and α7-nAChR expression induced by immobilization was similar to that caused by denervation and sepsis. When there is deprivation of neural influence or activity, such as sepsis, burn, or denervation, the immature γ-nAChR and the neuronal α7-nAChR, are re-expressed in skeletal muscle. Each of them has different electrophysiological characteristics and affinity for agonists, and it might play a special role in maintaining neurotransmission [32–34].

EA as a therapeutic intervention is widely used in the United States and around the world^6^. Previous studies have demonstrated that acupuncture increases the blood flow in skeletal muscle [35, 36] and evokes somatosensory responses of the brain, spinal cord and muscle [37], resulting in a reduction of muscle fatigue. They all have obvious improvement in muscle atrophy, but the mechanism of muscle atrophy is still unclear [38, 39]. In the current study, we determined that EA ameliorates muscle wasting in hind-limb immobilization rats and attenuates γ- and α7-nAChR re-expression, increasing the NRG-1 expression.

NRG-1 is a key factor in increased transcription of acetylcholine receptors (AChRs) and MuSK and regulates AChRs clusterization [40]. Axonal NRG-1 regulates myelin sheath thickness, which considered a biochemical measure of myelin sheath thickness [41]. Western blot analysis shows the expression of NRG-1 significantly decreased compared with that in the control group. This result demonstrated that immobilization induces demyelination of nerves, which is concordant with the Electromyography(EMG)findings.

In this study, expressions of NRG-1 decreased after immobilization, which could reduce the inhibitory signals, thereby activating γ- and α7-nAChR expression. What is more, increases in the expression of NRG-1 were observed in the EA group, These results may illustrate that EA was effective in improving the proliferation of AChRs through regulating NRG-1, leading to muscle fiber regeneration.

Several limitations exit in this study. Firstly, the upstream and downstream pathways associated with EA and NRG-1 expression are not investigated in this experiment. It is possibly associated with the NRG/ErbB signaling pathway. Secondly, this study revealed the close relation between the EA and γ- , α7-nAChR changes in immobilization, but the direct causal role of γ- and α7-nAChR in immobilization need to be validated with genetic manipulations. Further studies are, however, needed to more comprehensively explore this hypothesis.

In summary, the present findings highlight that immobilization decrease the neuromuscular function and activate fetal nAChR (γ-nAChR) and neuronal α7-nAChR (α7-nAChR) expression. The activation of γ- and α7-nAChR is associated with the reduction of NRG-1. EA suppressed the expression of γ- and α7-nAChR, increased the NRG-1 expression level and improved the neuromuscular function in skeletal muscles. These findings suggest EA is an effective non-pharmacological intervention to alleviate neuromuscular dysfunction and ameliorate skeletal muscle atrophy.

## MATERIALS AND METHODS

### Animals and groups

Male adult Sprague-Dawley (SD) rats (weight range: 240–260g) were acquired from the Experimental Animal Center of Chongqing Medical University (Chongqing, China) and housed in a controlled environment (25 ± 2°C, 60% humidity and 12:12-h light-dark cycle) for one week with free access to food and water. Based on the Animal Care and Use Committee of Chongqing Medical University, all rats received humane care. The rats were randomly divided into four groups: Control, Sham (Sham group was sham-immobilized by insertion of the hypodermic needles into the bone, which were removed immediately thereafter), Immobilization (Immobilized group was immobilized in one hind limb for a period of 14 days by pinning of knee and ankle joints at 90 degrees using hypodermic needles), and EA (Electroacupuncture treatment was performed for 20 minutes every day for 14 days along with immobilization) (n=14/group, there were 8 rats used for neuromuscular function observations and the other 6 were used for molecular biological studies). The experiment was approved by the Institution Animal Care and Use Committee (IACUC) and carried out according to the Animal Experimentation Regulations of Chongqing Medical University.

### Surgical procedures

The pining-immobilization model, previously described and used in many studies, was used for the current studies [22, 28]. After 1 week of acclimatization, immobilization procedure was performed. The rats were anesthetized with pentobarbital (60–70 mg/kg intraperitoneal (i.p.) injection). The knee and ankle joints were immobilized, respectively, by inserting 25-gauge hypodermic needles through the proximal tibia into the distal femur to produce 90° flexion at the knee, and 27-gauge hypodermic needles through the calcaneus into the distal tibia to fix the ankle joint at 90°. The sham immobilized limb was subjected to the same manipulations, including boring a hole through the joints but excluding pin insertion to immobilize the joints.

### Electroacupuncture (EA) treatment

EA treatment is administered for 14 days along with immobilization. The rats were kept in specially designed restraint so that they would remain in a recumbent position during EA treatment. EA was performed for 20 minutes every day for 14 days. Acupuncture points were selected according to the WHO Standard Acupuncture guidelines [42]. The positive point (Huan Tiao, GB30) located at the posterior upper border of the hip joint of the hind limbs, vertical needling to a depth of 6mm. The negative point (Zu San Li, ST36) is 5mm beneath the capitulum fibulae and 2mm lateral to the knee-joint about 7mm deep. The needles were connected to an SDZ-II Electronic acupuncture instrument with a 20Hz electric frequency using continuous wave, electric current 1mA. Disposable sterile needles with a diameter of 0.25 mm (Hua Tuo Medical & Health Material Co.Ltd. Wuhan, China) were used.

### Evaluation of neuromuscular function

Electromyography (EMG) recordings were measured before and after the immobilization. The data were obtained from the right sciatic nerve that was stimulated supramaximally (intensity 3 V, duration 0.2 ms, and frequency 1 Hz) using a direct stimulation electrode (BL-420F; Systems, Inc, Chengdu, China) on the sciatic nerve. Compound muscle action potential (CMAP) was recorded using a superficial disc electrode located on the tibialis anterior muscle before and after immobilization, as described previously [43]. The data were analyzed using the BL-420F USB2.0S (I) version 1.0.2 software Chengdu TaiMeng Instrument Company, Chengdu, China), with amplitude, latency, and duration of CMAP as the primary parameters. The Nerve conduction velocity (NCV) was calculated as the distance of conduction and/or latency time. Neuromuscular dysfunction was defined as a decrease by ≥20% of the baseline in the CMAP amplitude [44, 45]. Twitch tension was evaluated using evoked mechano- myography with a nerve stimulator (BL-420F; Systems, Inc, Chengdu, China) along with a force transducer. The twitch tension values of the respective tibialis muscle were recorded via an amplifier and displayed using the BL-420F USB2.0S (I) version 1.0.2 software. Anesthesia was maintained with supplemental intermittent doses of pentobarbital 10–20 mg/kg i.p. injection, empirically administered every 10–20 min. The temperature of rats was maintained at 35.5°–37°C with a heat lamp.

### Tissue preparation

Before the collection of tissues, all rats were sacrificed by deep anaesthesia with sodium pentobarbital (65 mg /kg i.p. injection). Body and muscle wet weights were measured shortly after death, the tibialis anterior muscle specimens of rats were collected in 4% paraformaldehyde or frozen with liquid nitrogen.

### Muscle fiber cross-sectional area and muscle fiber size

All samples were cut into 8μm-thick sections using the LEICA (Wetzlar, Germany, RM2135), alcohol dewaxing and hydration were performed for hematoxylin-eosin (HE) staining. Images were visualized with an Olympus BX51 inverted microscope with a Photonic Science CCD camera (Olympus DP50, Tokyo, Japan). Image-Pro Plus Image (IPP, Version 6.0, Media Cybernetics, USA) software was used to measure the cross sectional area of the muscle fibers and muscle fiber size.

### Western blot analysis for α7-nAChR, γ-nAChR and neuregulin-1 protein expression

Tissues were homogenized using lysis buffer (Beyotime, China), and supernatants were collected followed by 5min centrifugation of the tissue homogenate at 10,000 rpm at 4°C. After quantitative analysis of protein concentration, approximately 40 ug of the sample was loaded in each lane for SDS-PAGE electrophoretic separation, transferred to polyvinylidene fluoride membranes (Millipore, Billerica, MA, USA), blocked with 5% skim milk in Tris-buffered saline for 1 h at 37.8 °C, and then incubated overnight at 4°C with sc-13998 (Santa Cruz, Inc. dilution: 1:500), ab10096 (1:1000), or an anti-neuregulin (NRG-1) antibody (dilution 1:200, sc-28916, Santa Cruz) as primary antibodies. The membranes were incubated with secondary antibody (1:2000; Beyotime) at room temperature for 2h. Chemiluminescence Imaging System (Bio-Rad Co. USA) was used to take photos for the bands. All results were normalized to glyceraldehyde-3-phosphate dehydrogenase (GAPDH) levels.

### Immunofluorescence staining for α7-nAChR and γ-nAChR

An immunofluorescence assay was performed to identify the expression of α7- and γ-nAChR in muscles. The endogenous peroxidase was inactivated by incubating the tissue sections in 3% hydrogen peroxide for 30min at 25 °C. The antigen was retrieved using sodium citrate buffer (0.01mol / L, pH 6.0) at 96-98°C for 10 min; Then, the muscle sections were incubated with 10% normal goat serum (BosTER Biological Company, Wuhan, China) for 30min at room temperature. Then, the α7-nAChR antibody (ab10096, Abcam Ltd. dilution 1:800) and γ-nAChR antibody (sc-13998, Santa Cruz, Inc., dilution: 1:500) were incubated with the samples overnight at 4°C. After washing in phosphate-buffered saline, these sections were incubated with fluorescein isothiocyanate-conjugated Goat anti-rabbit IgG antibody to stain for α7-nAChR (dilution 1:200; #A23320 Abbkine, Inc, California, USA) or Goat anti-rabbit IgG antibody for γ-nAChR (dilution 1:200; #A23220 Abbkine, Inc, California, USA) for 1h at room temperature to detect each primary antibody. Images were acquired using a Nikon A1^+^ R microscope (Nikon, Inc, Tokyo, Japan).

### Statistical analyses

If data were normally distributed the results are displayed as means±SD, otherwise as median and the respective 25% and 75% quartiles. Significant differences between the groups were analyzed using One-way analysis of variance followed by Tukey’s post hoc test if the data was normally distributed. For data with other distributions Kruskal-Wallis followed by a post-hoc test according to Bonferoni method was used. Differences with *P* values < 0.05 were considered statistically significant. CMAP data are also analyzed by principal component analysis (PCA) and hierarchical cluster analysis (HCA). The data set is standardized to remove the effect of scaling differences between parameters with unit variance-scaled. PCA and HCA were performed with SIMCA-P software (version13.0, Umetrics, Sweden). All analysis was performed using the statistical software package SPSS 17.0 (IBM Corp, Armonk, NY).

To illustrate the position of the single units in relation to each other, a principal component analysis (PCA) was performed. PCA projects the units from the multidimensional space to a space of lower dimensionality while attempting to preserve the distance relations between them. The first principal component (PC1) accounts for as much of the variability in the data as possible and the PC2 accounts for the next highest levels of variation in declining order. This method also indicates which set of parameters best explains the dissimilarity between groups. To test whether theneuromuscular function of rats can be divided into groups based on CMAP data, HCA was also employed with standardized data set.
